# Extensive and persistent tongue ulceration is an early character of dyskeratosis congenita

**DOI:** 10.1186/s13023-025-03721-4

**Published:** 2025-04-21

**Authors:** Xuefeng Zhang, Hongxia Dan, Yu Zhou, Wanxin Sun, Wanchun Yang, Xin Zeng

**Affiliations:** 1 State Key Laboratory of Oral Diseases, National Center of Stomatology, National Clinical Research Center for Oral Diseases, West China Hospital of Stomatology, Sichuan University, Chengdu, 610041 Sichuan China; 2 Department of Neurosurgery, West China Hospital, Sichuan University, Chengdu, 610041 Sichuan China

**Keywords:** Dyskeratosis congenita, Tongue ulceration, *DKC1*

## Abstract

**Background:**

Dyskeratosis congenita (DC) is a rare and fatal disease, presenting with a classic triad of skin pigmentation, nail dystrophy and oral leukoplakia. However, diagnosing DC is challenging based solely on the protean manifestations and multisystemic involvement. Therefore, it is urgent to identify an early feature facilitating initial suspicion of DC.

**Results:**

In this study, we enrolled a cohort of six male children diagnosed with DC, all of whom exhibited erosions or ulcers on the tongue, while five of them did not display the complete classic triad. Strikingly, oral erosions or ulcers have never been included in any existing clinical diagnostic criteria for DC. Through a retrospective analysis, we further demonstrated that extensive and persistent tongue ulceration emerges as an early and practicable clinical marker, provoking suspicion of DC even in the absence of the classic triad.

**Conclusions:**

Our findings challenge prevailing diagnostic criteria and advocates for an expanded consideration of tongue ulceration as a primary and indicative manifestation of DC, thereby affording a strategic advantage for early detection and intervention of this lethal disease.

**Supplementary Information:**

The online version contains supplementary material available at 10.1186/s13023-025-03721-4.

## Introduction

Dyskeratosis congenita (DC) is fatal disease marked by multisystemic involvement with an incidence of 1/100, 000. DC presents with a triad of skin pigmentation, nail dystrophy and oral leukoplakia (OLK) [1]. The primary causes of mortality in DC are bone marrow failure (BMF), pulmonary disease and malignancies [2, 3]. In most cases, DC presented over the first two decades of life [4], and the median age of DC diagnosis is 15 years [5]. However, progressive disease features may appear before the age of 10 [6], and some patients even present with fatal complications in early childhood. Given the early onset and poor outcomes, timely suspicion and diagnosis are significant to improve intervention strategies and prognosis.

In clinical practice, suspicion of DC guides the implementation of telomere measurement and targeted genetic testing, facilitating the definitive confirmation. The minimal clinical criteria for DC require the presence of at least two of the four major features, including abnormal skin pigmentation, nail dystrophy, OLK and BMF, as well as two or more somatic features [3, 7]. However, suspecting DC based solely on classic triad is challenging and often leads to delayed detection [7]. Some DC patients had none of the classic triad of DC [3, 8–11]. Thus, there is a pressing need of feasible manifestations for DC, which facilitates initial suspicion at early stages.

During our clinical practice, we noticed that DC patients at our institution had tongue ulcers or erosions, but not having the complete classic triad at first visits. Notably, oral ulcers or erosions have never been included in any existing clinical diagnostic criteria for DC [3, 7, 12, 13]. Based on these findings, we conducted a comprehensive investigation to determine whether oral ulceration could potentially serve as an early character for suspecting DC compared with the classic mucocutaneous triad.

## Patients and methods

A literature search was conducted in PubMed, using the keywords ‘Dyskeratosis congenita’, to identify relevant English-language publications up until December 2022. The initial search yielded a total of 446 cases, which were subjected to rigorous evaluation to ensure medical accuracy. Two independent medical doctors meticulously reviewed the full texts of the retrieved articles, and a third doctor subsequently examined the analysis for consistency. Inclusion criteria for the analysis encompassed confirmed cases, while reports lacking unequivocal data on onset and diagnosis, as well as duplicate reports of the same case(s), were excluded.

Furthermore, six patients with DC were enrolled in West China Hospital of Stomatology, Sichuan University, and currently followed. Finally, a cohort of 452 DC patients was assembled, and a detailed analysis was conducted on the ages of diagnosing DC, the presence of the classic triad and genetic mutations. The presence of oral ulcerations was examined comprehensively, including an exploration of their ages and locations, while also establishing correlations with the classic triad.

Statistical analyses were carried out using R (version 4.0.3 software), and a significance level of *p* < 0.05 was defined to determine statistical significance.

## Results

### Oral ulceration as an independent entity of DC: insights from two cases

Although oral ulceration has never been included in any existing clinical diagnostic criteria for DC, we are considering whether oral ulceration could potentially serve as an independent and early feature of DC, and whether patients with oral ulceration would progressively develop the classic triad. To address these questions, we focused on two representative cases within our cohort.

**Fig. 1:**
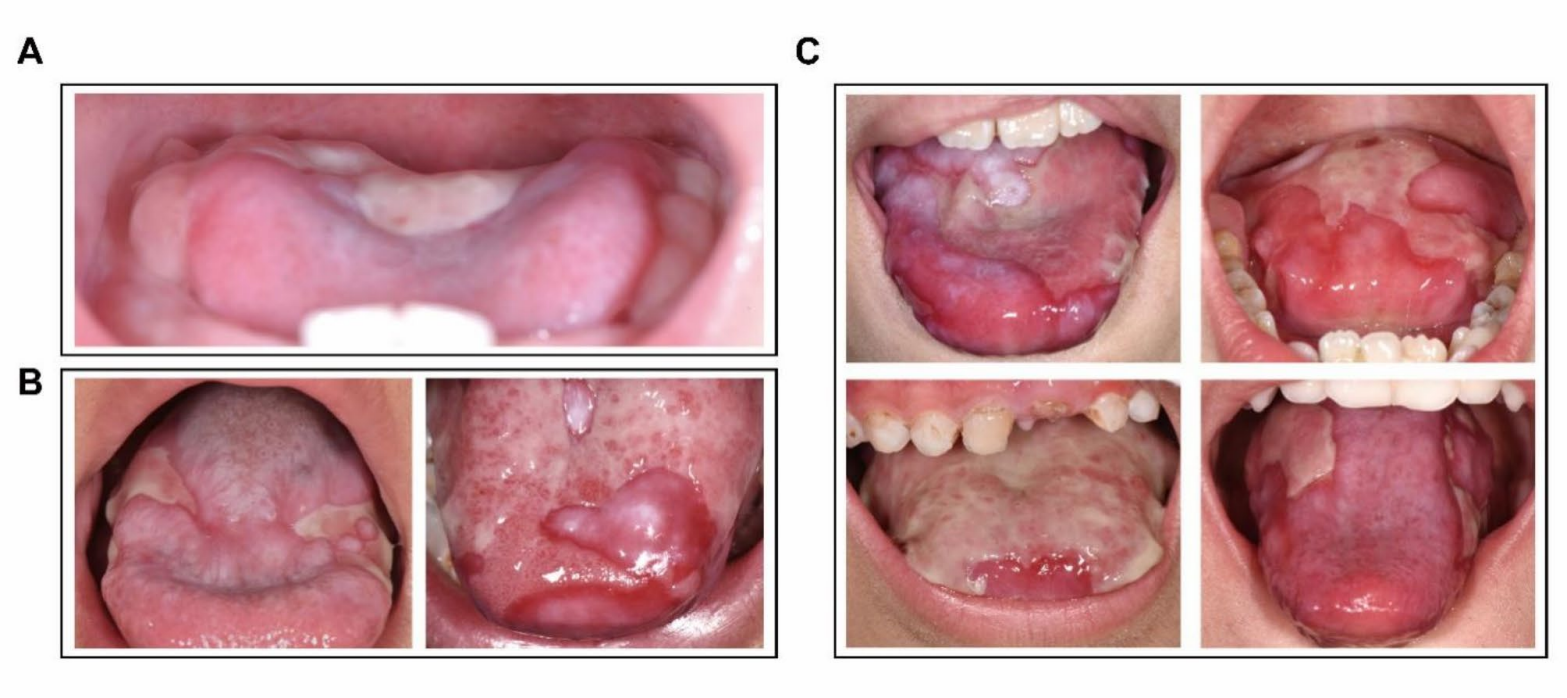
Extensive oral ulceration in DC patients. **(A)** Images of tongue ulcers in a male DC patient at the age of 1 year and 2 months (Case 1). **(B)** Images of tongue erosions at the age of 4 years (left panel) and oral leukoplakia (OLK) at the age of 9 years (right panel) of a male DC patient (Case 2). **(C)** Images of tongue ulcers or erosions in other four cases of DC

Case one involved a male patient of 1 year and 2 months old, who presented with 7-month ulceration on the dorsum and right side of the tongue. Oral examination revealed a sizable erosive surface on the dorsum of the tongue and both sides of the abdomen, with no other mucocutaneous abnormalities (Fig. 1A). Genetic testing indicated a mutation in the *DKC1* gene. qPCR analysis revealed the decreased telomere lengths of the patient, compared to the mother and a volunteer. Thus, a diagnosis of DC was made. This case suggests that oral ulceration may represent an independently early character of DC, separating from all the classic triad.

Case two involved a 4-year-old male patient in 2018 presented with whiteness and erosion of the lingual dorsum and bilateral lingual margins for one year. The original erosion lesion surface was partially covered with a pearlescent white pattern, and the erosion on both sides of the abdomen of tongue did not improve (Fig. 1B, left panel). There was hyperpigmentation on the posterior part of both buccal mucosae, and no other oral mucosa abnormalities were observed. Genetic testing revealed a pure-sibling mutation in *DKC1*, leading to a diagnosis of DC. During the recent follow-up in 2023, we found emergence of typical OLK in oral cavity (Fig. 1B, right panel). This case suggests a potential progressive development from oral ulceration to OLK in oral lesions. Finally, we presented typical oral ulceration images of other four DC patients in our cohort, indicating that extensive and persistent ulceration is an early character of DC (Fig. 1C).

### Oral ulceration as a feasible and early character for initial suspicion of DC

To better identify the initial manifestations of DC, we conducted a comprehensive review of the published literature and six DC patients from our cohort, with specific focus on oral ulceration.

**Fig. 2:**
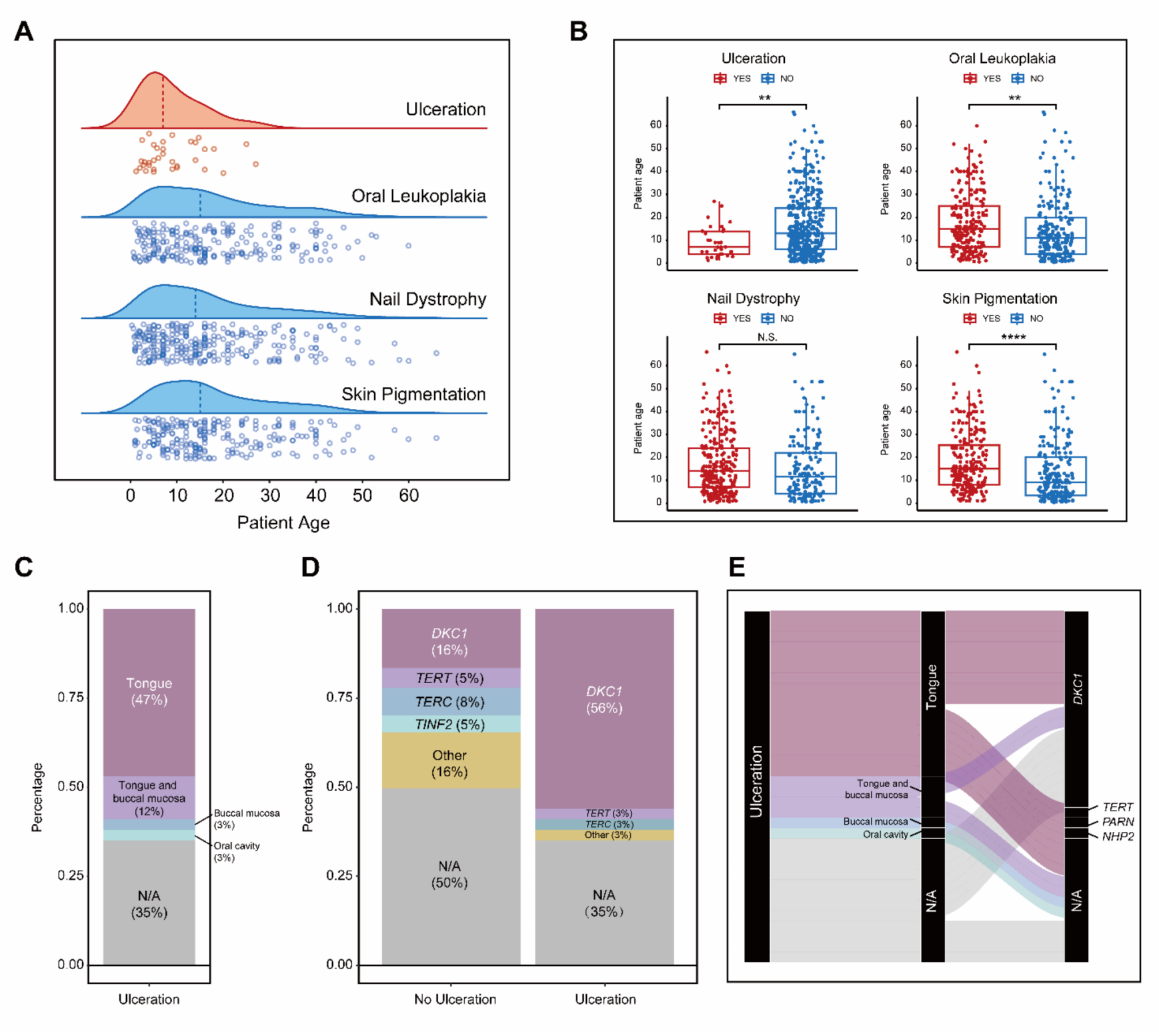
Tongue ulceration as an early character of DC. **(A)** Diagrams showing the distribution of ulceration and mucocutaneous triad related to patient ages. The dashed lines indicate the median ages. **(B)** Diagrams demonstrating that DC patients with oral ulceration exhibited significantly younger ages compared with those without oral ulceration, whereas the mucocutaneous triad exhibited an opposite trend. **(C)** Diagrams showing the distribution of ulceration sites in DC patients with oral ulceration, with the tongue being the most predominant location. **(D)** Diagrams showing the distribution of genetic mutations in DC patients, with a significantly higher proportion of *DKC1* mutations observed in DC patients with oral ulceration. **(E)** Sankey diagrams showing that the predominant site of ulceration in DC patients was the tongue, and the majority of these tongue ulceration patients carried *DKC1* mutations

In the cumulative group of 452 patients reported having DC (including six patients from our cohort), we identified a total of 34 DC patients with oral ulceration. Interestingly, unlike the scattered distribution of the classic triad with age, the character of ulceration was predominantly observed in children aged 0–10 years, and all reported cases of DC with oral ulceration were below the age of 30, which was earlier than the classic triad (Fig. 2A). Moreover, patients reported to have oral ulceration were significantly younger than those without oral ulceration, while patients reported to have OLK and skin pigmentation were older than those without OLK and skin pigmentation (Fig. 2B). Additionally, the presence or absence of nail dystrophy did not show a difference in age (Fig. 2B). All these facts support a notion that oral ulceration is an early character among the feasible manifestations of DC.

### Localization of oral ulceration and its association with *DKC1* mutations in DC

Next, we sought to investigate oral locations of ulceration. Results showed that among all the 34 cases, 47%patients exhibited ulceration localized on the tongue, and 12% cases with ulceration on both the tongue and buccal mucosa. Additionally, 3% cases reported ulceration occurrence on the buccal mucosa and oral cavity, respectively. Due to incomplete records in the literature, the ulceration sites of 35% patients remained unclear (Fig. 2C). The occurrence of DC is closely associated with various mutations in telomerase-related genes, like *DKC1*. Remarkably, it was observed that the incidence of *DKC1* mutations was significantly higher in DC patients with oral ulceration (56%) than those without oral ulceration (16%) (Fig. 2D). Due to the complexity of DC pathogenesis, diverse clinical presentations and variations in diagnostic criteria, there was a lack of genetic information in many cases. Notably, it revealed that the majority of oral ulceration occurred on the tongue, which was closely associated with *DKC1* mutations (Fig. 2E), suggesting a potential correlation between tongue ulceration and *DKC1* mutations in DC patients.

## Discussion

The clinical spectrum of DC at presentation is broad, since the infiltration of different organs leads to the development of protean manifestations. Current literature identified abnormal skin pigmentation, nail dystrophy and OLK as the most common manifestations of DC. While the successive manifestations can occur over years, the clinical picture may be a slowly forming mosaic. In order to characterize the clinical manifestations of DC, we conducted a systematic review of published literature and included data from six patients in our cohort. Our findings indicate that extensive and persistent ulceration on the tongue even without the classic triad, represents a feasible character of DC, thereby facilitating initial suspicion and intervention strategies at early stages.

DC is characterized by the presence of the triad of skin pigmentation, nail dystrophy and OLK [3, 14], with clinical suspicion primarily based on recognizing these manifestations for nearly a century [14–17]. However, tongue ulceration may serve as an earlier and feasible clinical feature for DC suspicion. Previous reports have noted tongue ulceration in patients with DC [18, 19], and in 2019, we reported a DC case presenting with persistent tongue ulceration instead of typical OLK [20]. Despite updates in diagnostic criteria, oral ulceration remains unmentioned, even among the minor features of the disease. In light of these issues, our study aimed to identify ulceration as an ideal early manifestation and serves as a feasible character aiding in the early suspicion of DC. The diagnostic potential of tongue ulceration in DC is noteworthy for several reasons. Firstly, tongue ulceration emerges earlier compared with any classic triad for initial suspicion of DC. Secondly, tongue ulceration exhibits high specificity and can be distinguished from other mucosal manifestations, preventing misinterpretation as OLK, white keratotic lesions, white patches or lichenoid changes [21, 22]. Thirdly, given the wide range of clinical presentations in DC and the lack of definitive laboratory tests, particularly in undeveloped regions, the presence of pain associated with tongue ulceration captures the attention of both patients and physicians, facilitating early detection. Last but important, our findings demonstrate that tongue ulceration may serve as a specific indicator of the major subtype of DC with *DKC1* mutation [3].

Mechanistically, DC carried mutations in genes such as *DKC1* leading to decreased telomerase activity and shortened telomeres, elucidating its genetic basis [7, 23–25] and linking it to telomere biology [26]. Notably, mutations in *DKC1* not only impair telomerase assembly and function but also compromise the pseudouridylation of ribosomal RNA. This dual impact underscores the severe phenotypes observed in X-linked DC cases. Tongue ulceration, as observed in our study, may result from heightened cellular turnover demands in rapidly regenerating mucosal tissues, exacerbated by impaired telomerase activity. Additionally, our studies highlight the role of other telomere-associated genes in modulating DC phenotypes. Variants in *TERT*, *PARN* and *NHP2*, are associated with severe disease subtypes, mirroring the effects of *DKC1* mutations [12, 27]. These genetic mutations further destabilize telomeres and compromise DNA repair mechanisms, contributing to persistent tissue damage such as mucosal ulceration.

## Conclusions

In sum, our study highlights the clinical significance of tongue ulceration as an early manifestation of DC, suggesting that extensive and persistent tongue ulceration even in the absence of the classic triad or BMF, can raise suspicion of DC, enabling early detection and intervention.

## Electronic supplementary material

Below is the link to the electronic supplementary material.

Supplementary Material 1

## Data Availability

All data and analysis can be acquired from corresponding authors upon reasonable request.

## Abbreviations

DC: Dyskeratosis congenita
OLK: Oral leukoplakia
BMF: Bone marrow failure
HH: Høyeraal-Hreidarsson

## References

[1] Dokal I. A disease of premature ageing. Lancet. 2001;358:S27. 11784576 10.1016/s0140-6736(01)07040-4

[2] Dokal I. Dyskeratosis congenita in all its forms. Br J Haematol. 2000;110(4):768–79. 11054058 10.1046/j.1365-2141.2000.02109.x

[3] Dokal I. Dyskeratosis congenita. Stiehm’s Immune Deficiencies. 2014:267– 80.

[4] Fogarty PF, Yamaguchi H, Wiestner A, Baerlocher GM, Sloand E, Zeng WS, et al. Late presentation of dyskeratosis congenita as apparently acquired aplastic anaemia due to mutations in telomerase RNA. Lancet. 2003;362(9396):1628–30. 14630445 10.1016/S0140-6736(03)14797-6

[5] Savage SA, Alter BP. Dyskeratosis congenita. Hematol Oncol Clin N Am. 2009;23(2):215–31. 10.1016/j.hoc.2009.01.003 PMC2702847 19327580

[6] Walne AJ, Marrone A, Dokal I. Dyskeratosis congenita: a disorder of defective telomere maintenance? Int J Hematol. 2005;82(3):184–9. 16207588 10.1532/IJH97.05067

[7] Dokal I, Vulliamy T, Mason P, Bessler M. Clinical utility gene card for: dyskeratosis congenita - update 2015. Eur J Hum Genet. 2015;23(4). 10.1038/ejhg.2014.170 PMC4667501 25182133

[8] Sznajer Y, Baumann C, David A, Journel H, Lacombe D, Perel Y, et al. Further delineation of the congenital form of X-linked dyskeratosis congenita (Hoyeraal-Hreidarsson syndrome). Eur J Pediatr. 2003;162(12):863–7. 14648217 10.1007/s00431-003-1317-5

[9] Lim BC, Yoo SK, Lee S, Shin JY, Hwang H, Chae JH, et al. Hoyeraal-Hreidarsson syndrome with a DKC1 mutation identified by whole-exome sequencing. Gene. 2014;546(2):425–9. 24914498 10.1016/j.gene.2014.06.011

[10] Xin ZT, Beauchamp AD, Calado RT, Bradford JW, Regal JA, Shenoy A, et al. Functional characterization of natural telomerase mutations found in patients with hematologic disorders. Blood. 2007;109(2):524–32. 16990594 10.1182/blood-2006-07-035089

[11] Alebrahim M, Akateh C, Arnold CA, Benissan-Messan D, Chavez JA, Singh N, et al. Liver transplant for management of hepatic complications of dyskeratosis congenita: A case report. Exp Clin Transpl. 2022;20(7):702–5. 10.6002/ect.2020.0073 PMC8155112 33272154

[12] Savage SA, Bertuch AA. The genetics and clinical manifestations of telomere biology disorders. Genet Med. 2010;12(12):753–64. 21189492 10.1097/GIM.0b013e3181f415b5 PMC3825100

[13] Shimamura A, Alter BP. Pathophysiology and management of inherited bone marrow failure syndromes. Blood Rev. 2010;24(3):101–22. 20417588 10.1016/j.blre.2010.03.002 PMC3733544

[14] Zinsser F. Atropha cutis reticularis pigmentation, dystrophia ungiumet leukoplakia Oris. Ikonogr Dermatol (Hyoto). 1906;5:219–23.

[15] Engman M. A unique case of reticular pigmentation of the skin with atrophy. Arch Derm Syph. 1926;13:685–7.

[16] Cole H, Rauschkolb J, Toomey J. Dyskeratosis congenita with pigmentation, dystrophia Unguis and leukokeratosis Oris. Archives Dermatology Syphilology. 1930;21(1):71–95. 10.1001/archderm.1955.01540280027005 14360753

[17] Kelmenson DA, Hanley M. Dyskeratosis congenita. N Engl J Med. 2017;376(15):1460. 28402761 10.1056/NEJMicm1613081

[18] Walter JE, Armanios M, Shah U, Friedmann AM, Spitzer T, Sharatz SM, et al. Case records of the massachusetts general hospital. Case 41-2015. A 14-Year-Old Boy with immune and liver abnormalities. N Engl J Med. 2015;373(27):2664–76. 26716919 10.1056/NEJMcpc1408595

[19] Hreidarsson S, Kristjansson K, Johannesson G, Johannsson J. A syndrome of progressive pancytopenia with microcephaly, cerebellar hypoplasia and growth failure. Acta Paediatr. 1988;77(5):773–5. 10.1111/j.1651-2227.1988.tb10751.x 3201986

[20] Zhang X, Wang J, Wang F, Jin X, Zhou Y, Dan H, et al. Extensive erosion instead of leukoplakia can be the oral manifestation of dyskeratosis congenita. Oral Dis. 2019;25(3):919–21. 30620099 10.1111/odi.13035

[21] AlSabbagh MM. Dyskeratosis congenita: a literature review. J Dtsch Dermatol Ges. 2020;18(9):943–67. 32930426 10.1111/ddg.14268

[22] Nico MMS, Yendo TM, Lourenco SV. Understanding oral mucosal lesions in dyskeratosis congenita: from interface inflammation to squamous cell carcinoma, an observational report. Int J Dermatol. 2022;61(10):e366–8. 35388911 10.1111/ijd.16215

[23] Agarwal S, Loh Y-H, McLoughlin EM, Huang J, Park I-H, Miller JD, et al. Telomere elongation in induced pluripotent stem cells from dyskeratosis congenita patients. Nature. 2010;464(7286):292–6. 20164838 10.1038/nature08792 PMC3058620

[24] Batista LF, Pech MF, Zhong FL, Nguyen HN, Xie KT, Zaug AJ, et al. Telomere shortening and loss of self-renewal in dyskeratosis congenita induced pluripotent stem cells. Nature. 2011;474(7351):399–402. 21602826 10.1038/nature10084 PMC3155806

[25] Niewisch MR, Giri N, McReynolds LJ, Alsaggaf R, Bhala S, Alter BP, et al. Disease progression and clinical outcomes in telomere biology disorders. Blood. 2022;139(12):1807–19. 34852175 10.1182/blood.2021013523 PMC8952184

[26] Mitchell JR, Wood E, Collins K. A telomerase component is defective in the human disease dyskeratosis congenita. Nature. 1999;402(6761):551–5. 10591218 10.1038/990141

[27] Ballew BJ, Savage SA. Updates on the biology and management of dyskeratosis congenita and related telomere biology disorders. Expert Rev Hematol. 2013;6(3):327–37. 23782086 10.1586/ehm.13.23

