# Supplementary material for: Extensive and persistent tongue ulceration is an early character of dyskeratosis congenita
Source: Orphanet J Rare Dis. 2025 Apr 21;20:192. doi: 10.1186/s13023-025-03721-4 (PMC12012981; doi:10.1186/s13023-025-03721-4)
Supplement: Supplementary file 1 — Supplementary Material 1 [file 13023_2025_3721_MOESM1_ESM.docx]

**Point-To-Point Responses to Reviewer's Comments**

**Reviewer reports:**

The manuscript is interesting and relevant. I would like to suggest the authors to focus in the discussion on genetic and molecular mechanisms and skin the hystorical informations.

**Response:**

We appreciate your positive comments that you find our work interesting and relevant. We also sincerely appreciate your thoughtful and constructive suggestion on the improvement of the Discussion section.

In response to your suggestion, we have revised the Discussion section to focus more on the genetic and molecular mechanisms underlying dyskeratosis congenita (DC) and have minimized the historical information. Specifically, we have expanded on the roles of mutations in genes such as *DKC1*, and how genetic alterations lead to decreased telomerase activity, telomere shortening, and compromised DNA repair mechanisms. We have also discussed how these molecular defects contribute to the clinical manifestations of DC, including persistent tongue ulceration observed in our study. All these improvements have been added in Discussion of revised manuscript.

Finally, we thank again for your rigorous review that significantly improves our manuscript.
